# STING and cGAS gene expressions were downregulated among HIV-1-infected persons after antiretroviral therapy

**DOI:** 10.1186/s12985-021-01548-6

**Published:** 2021-04-15

**Authors:** Lorena Leticia Peixoto de Lima, Allysson Quintino Tenório de Oliveira, Tuane Carolina Ferreira Moura, Ednelza da Silva Graça Amoras, Sandra Souza Lima, Andrea Nazaré Monteiro Rangel da Silva, Maria Alice Freitas Queiroz, Izaura Maria Vieira Cayres-Vallinoto, Ricardo Ishak, Antonio Carlos Rosário Vallinoto

**Affiliations:** Laboratory of Virology, Institute of Biological Sciences, Federal University of Pará (UFPA), Belém, Pará Brazil

**Keywords:** STING, CGAS, IFN-α, Expression, HIV-1, ART

## Abstract

**Background:**

The HIV-1 epidemic is still considered a global public health problem, but great advances have been made in fighting it by antiretroviral therapy (ART). ART has a considerable impact on viral replication and host immunity. The production of type I interferon (IFN) is key to the innate immune response to viral infections. The STING and cGAS proteins have proven roles in the antiviral cascade. The present study aimed to evaluate the impact of ART on innate immunity, which was represented by STING and cGAS gene expression and plasma IFN-α level.

**Methods:**

This cohort study evaluated a group of 33 individuals who were initially naïve to therapy and who were treated at a reference center and reassessed 12 months after starting ART. Gene expression levels and viral load were evaluated by real-time PCR, CD4^+^ and CD8^+^ T lymphocyte counts by flow cytometry, and IFN-α level by enzyme-linked immunosorbent assay.

**Results:**

From before to after ART, the CD4^+^ T cell count and the CD4^+^/CD8^+^ ratio significantly increased (p < 0.0001), the CD8^+^ T cell count slightly decreased, and viral load decreased to undetectable levels in most of the group (84.85%). The expression of *STING* and *cGAS* significantly decreased (p = 0.0034 and p = 0.0001, respectively) after the use of ART, but IFN-α did not (p = 0.1558). Among the markers evaluated, the only markers that showed a correlation with each other were *STING* and CD4^+^ T at the time of the first collection.

**Conclusions:**

ART provided immune recovery and viral suppression to the studied group and indirectly downregulated the *STING* and *cGAS* genes. In contrast, ART did not influence IFN-α. The expression of *STING* and *cGAS* was not correlated with the plasma level of IFN-α, which suggests that there is another pathway regulating this cytokine in addition to the STING–cGAS pathway.

**Supplementary Information:**

The online version contains supplementary material available at 10.1186/s12985-021-01548-6.

## Background

Human immunodeficiency virus 1 (HIV-1) infection and acquired immune deficiency syndrome (AIDS) are public health problems due to their pandemic proportions. The virus infects about 37.9 million people worldwide, of which 24.5 million have access to antiretroviral therapy (ART) [1].

HIV-1 infects CD4^+^ T cells, which causes immunodeficiency characterized by the reduction in these cells counts and an increase in CD8^+^ cytotoxic T cells [2]. Since the discovery of AIDS, several efforts have been made to contain the spread of the virus and prevent the infected individual from developing severe immunodepression leading to death. Of all the strategies studied and applied to date, ART has had great success, and its main objective is to suppress long-term viral multiplication and preserve or restore immune function [3–5].

The innate immune response to infections is mainly based on the recognition of the so-called pathogen-associated molecular patterns (PAMPs) or damage-associated molecular patterns. Such recognition is only possible through the activity of pattern recognition receptors that are sensitive to signs of invasion by pathogenic microorganisms and cellular damage. When pattern recognition receptors recognize molecular patterns, they send a signal to stimulate the antiviral innate immune response and/or pro-inflammatory cytokine response. The action of the initial response is mainly represented by interferon (IFN)-I, macrophages, and natural killer cells [6–9].

Because nucleic acids are a key element in pathogen replication, they are one of the main groups of PAMPs recognized by Toll-like receptors, members of the RIG-1-like family [10], and the stimulator of interferon genes (STING), which was identified as a new nucleic acid detector [11]. STING binds directly to dsDNA or associates with second messengers called cyclic dinucleotides (CDNs), such as c-AMP, c-GMP, c-di-AMP, or c-di-GMP [12]. By binding to DNA or CDNs, STING is activated through the association with and activation of interferon regulatory factor (IRF) 3 and IRF7, after which it stimulates the transcription of innate immunity genes such as the IFN-I gene. In a second signaling cascade, STING also activates the nuclear factor (NF)-κB pathway, which leads to the production of pro-inflammatory cytokines [13, 14]. The activity of the cGMP-AMP synthase (cGAS) enzyme, which is responsible for the synthesis of the second messenger cGAMP, is essential for the detection of CDNs by STING and is therefore characterized as an essential element in the cytosolic signaling cascade by STING [15].

STING plays a major role in the production of IFN-I, as shown by both knockdown and overexpression experiments in different cells. Studies with STING-deficient animals show that they are viable but extremely sensitive to infection by a variety of DNA and RNA viruses [11, 13, 16]. Different viruses with DNA and RNA genomes have been implicated in cGAS/STING-dependent activation, including HIV-1 [17–19]. The silencing or deficiency of *cGAS* or *STING* strongly inhibits the induction of interferons and other cytokines. Cells with mutant *cGAS* are also unable to mount any detectable immune response against HIV infection [13, 19–22]. In addition, ART seems to interfere with *cGAS* activity, reducing IFN-I production [21, 22].

The present study evaluated the influence of ART on the expression of *STING* and *cGAS*, the production of type I IFN, and the levels of laboratory markers often used to monitor infection (CD4^+^, CD8^+^, and ratio of CD4^+^/CD8^+^ T cells and viral load) in a cohort infected with HIV-1.

## Methods

### Study population

Sixty-two HIV-1-infected patients were initially contacted, but only 48 were eligible to start the study because they were not taking therapy. Ethnicity was not a criterion for selecting and separating patients into subgroups, since the target population is representative of the population of Belem, capital of Para State, that is composed by interethnic mixture. Likewise, transmission modes were not evaluated.

The 48 HIV-1-infected individuals, admitted to the Serviço de Assistência Especializada Casa Dia (Casa Dia Specialized Care Service), located in the municipality of Belem, Para, Brazil, were over 18 years old, and of either sex. All the patients enrolled in the present study were recently diagnosed; but did not know how long they had been infected. They were followed up for an average of 12 months from the start of therapy. At the end of the 12-month follow-up, only 33 individuals were still using ART and returned to the reference center.

The project was approved by the Research Ethics Committee of the Center for Oncology Research at the Federal University of Pará (CAAE 31446920.0.0000.0018). All subjects were duly informed of the objectives of the study, and those who agreed to participate in the study signed an informed consent form.

### Sample collection and storage

Whole blood was collected in two 5-mL tubes containing K_3_-EDTA. The samples were placed in an appropriate container for conservation and transported to the Laboratory of Virology of the Institute of Biological Sciences of the Federal University of Pará (UFPA). A portion of each whole-blood sample was used for quantification of CD4^+^ and CD8^+^ T cells, and the other part was centrifuged for separation of plasma and cells. The plasma HIV-1 viral load and INF-α level were quantified, and leukocytes were stored after the addition of TRIzol to maintain RNA integrity. All samples were stored at − 70 °C until use.

### RNA extraction

Total RNA was extracted from peripheral-blood leukocytes using the TRIzol™ Plus RNA Purification Kit (ThermoFisher Scientific, Waltham, Massachusetts, USA), and all steps followed the protocol recommended by the manufacturer. The concentration of extracted RNA was determined using a NanoDrop™ fluorimeter (ThermoFisher Scientific, Waltham, Massachusetts, USA) according to the manufacturer's instructions. All total RNA samples were diluted to 50 ng/µL for complementary DNA (cDNA) synthesis.

### Reverse transcription

The extracted RNA was converted into cDNA using the High Capacity cDNA Reverse Transcription® with RNase Inhibitor kit (Applied Biosystems, Foster City, CA, USA). For the reverse transcription reaction, a mix of 20 µL was prepared, which contained 2 µL of 10 × RT Buffer, 0.8 µL of 25 × dNTP Mix (100 nM), 2 µL of random primer, 1 µL of MultiScribe™ Reverse Transcriptase, 1 µL of RNaseOUT™, and 3.2 µL of ultra-pure water, which were provided by the kit, plus 10 µL of extracted RNA. The mixture was placed in a Mastercycler Personal thermocycler (Eppendorf, Hamburg, Germany) and cycled at 25 °C for 10 min, 37 °C for 120 min, and 85 °C for 5 min.

### mRNA quantification by real-time quantitative PCR (qPCR)

Initially, the standardization of qPCRs with cDNAs and probes (endogenous genes and targets) was performed to calculate the efficiency of the amplification reactions. In the standardization reactions, different concentrations of cDNA (pure and in four twofold dilutions: 1:2, 1:4, 1:8, and 1:16) were tested. All reactions were performed in plates and in triplicate, and the same cDNA (at different dilutions) was analyzed at the same time as the different probes to construct an efficiency curve to validate the 2^−ΔΔCT^ computation method. All tests showed efficiency as expected (100% ± 10) [49].

The relative quantification of gene expression consisted of amplification of the target gene along with an endogenous normalization gene using TaqMan™ assays (Applied Biosystems, Foster City, CA, USA) and the StepOnePLUS™ Real-Time PCR System (Thermo Fisher Scientific, Waltham, MA, USA). The reactions were performed in singleplex format following the manufacturer's protocol. TaqMan Gene Expression Assays were used (Hs00736955_g1 for *STING*, Hs02786624_g1 for *cGAS*, and Hs02786624_g1 for the endogenous reference gene glyceraldehyde-3-phosphate dehydrogenase). The primer and probe sequences that make up the assays are not available by Thermo Fisher Scientific (Waltham, MA, USA). For the reaction, we used 15 µL of 2 × TaqMan® Universal PCR Master Mix, 1.5 µL of the 20 × TaqMan Gene Expression Assay, 3 µL of cDNA, and 10.5 µL of RNase-free water. The thermocycling conditions were 2 min at 50 °C, followed by 10 min at 95 °C and 1 min at 60 °C.

The relative quantification of target gene expression was calculated using the comparative CT method with the formula 2^−ΔΔCT^, where ∆∆C_t_ = ∆C_t_ sample − ∆C_t_ reference (Life Technologies, Carlsbad, CA, USA).

### CD4^+^ and CD8^+^ T cell counts

The CD4^+^ and CD8^+^ T cells were counted by flow cytometry (BD FACSCalibur™, Becton & Dickinson) with the FACSCount™ Reagents monitoring kit, following the protocol recommended by the manufacturer (Becton & Dickinson, San Jose, California, USA).

### Quantification of HIV-1 plasma viral load

The viral load was quantified by real-time PCR using the Sample Purific CV HIV-1 extraction kit (Abbott) and the HIV-1 viral load amplification kit (Abbott, Chicago, Illinois, USA). The units used were copies/mL converted by log_10_. Both the CD4^+^ and CD8^+^ T cells and the HIV-1 viral load were quantified according to the standard set by the National Network for the Determination of CD4^+^ and CD8^+^ T cells and Viral Load of the Department of HIV/AIDS and Viral Hepatitis of the Ministry of Health.

### Plasma quantification of IFN-I

The levels of IFN-I (IFN-α) were quantified in plasma samples with an IFN-α human enzyme-linked immunosorbent assay kit (Thermo Fisher Scientific, Waltham, MA USA) according to the manufacturer's recommendations.

### Data analysis

All information was entered into a database in Microsoft Excel. The evaluation of the frequency of viral load before and after ART was evaluated by the G test. The normality of numerical results was assessed using the Kolmogorov–Smirnov test. The T test or the Wilcoxon test was applied for the paired analysis of the variables CD4^+^ T cells, CD8^+^ T cells, CD4^+^/CD8^+^ T cell ratio, *STING* expression, *cGAS* expression, and IFN-α level. Pearson’s test or Spearman’s test was used for correlation analysis. All tests were performed using the programs GraphPad Prism 5.0 and BioEstat 5.0. The quadratic regression model was performed using MINITAB 14.0 software. Associations with p < 0.05 were considered significant.

## Results

The study group consisted mostly of males (77.08%), and the mean age was 32.6 years. Half of the individuals evaluated (50%) had at most a complete or incomplete secondary education, and 64.44% reported having a family income between 1 (R$ 1,100) and 3 minimum wages. Of the initial 48 individuals, only 33 made continuous use of antiretrovirals and could be evaluated after an average of 12 months of ART. For statistical purposes, the results of the paired samples in the first (without ART) and second collections (with ART) were used.

**Fig. 1:**
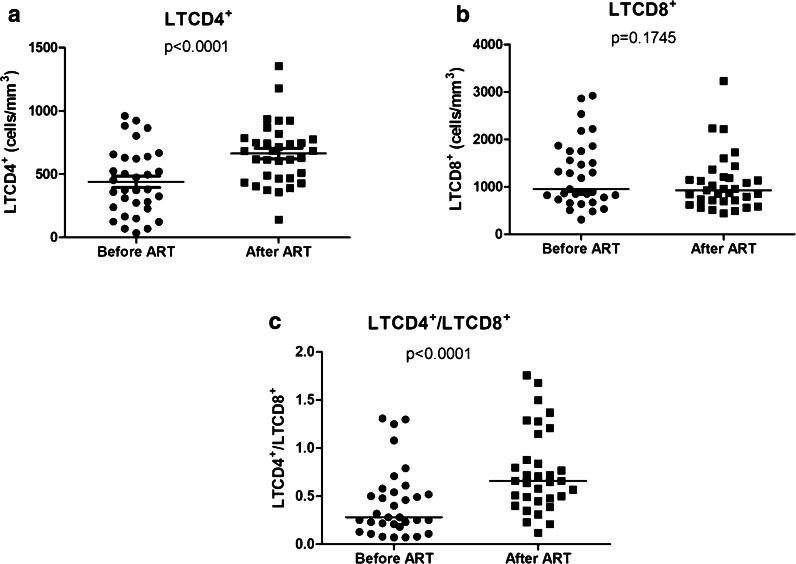
Evaluation of the levels of **a** CD4^+^ T cells, **b** CD8^+^ T cells, and **c** the CD4^+^/CD8^+^ T cell ratio before and after ART

In the evaluation of the CD4^+^ T cell count, a significant increase in the number of cells per mm^3^ was observed—from 439 to 662 cells/mm^3^ (p < 0.0001) (Fig. 1a). For CD8^+^ T cells, there was a slight reduction between the first and second collections (p = 0.1745), with medians of 956 and 928 cells/mm^3^, respectively (Fig. 1b). The CD4^+^/CD8^+^ T cell ratio increased significantly with the use of ART, with the median increasing from 0.28 to 0.66 (p < 0.0001) (Fig. 1c).

The HIV-1 viral load decreased between the first and second collections. Most individuals (84.85%) started to have an undetectable viral load (< 40 copies/mL) after using ART (Table 1).

**Table 1:** Quantification of HIV-1 viral load in individuals before and 12 months after ART

Viral load (copies/mL)	Before ART	After ART	p*
	n (%)	n (%)	
< 40	1 (3.03)	28 (84.85)	< 0.0001
41–999	3 (9.09)	3 (9.09)	
1000- 10,000	5 (15.15)	0 (0.0)	
> 10,000	24 (72.73)	2 (6.06)	

n; number of individuals; *G test

**Fig. 2:**
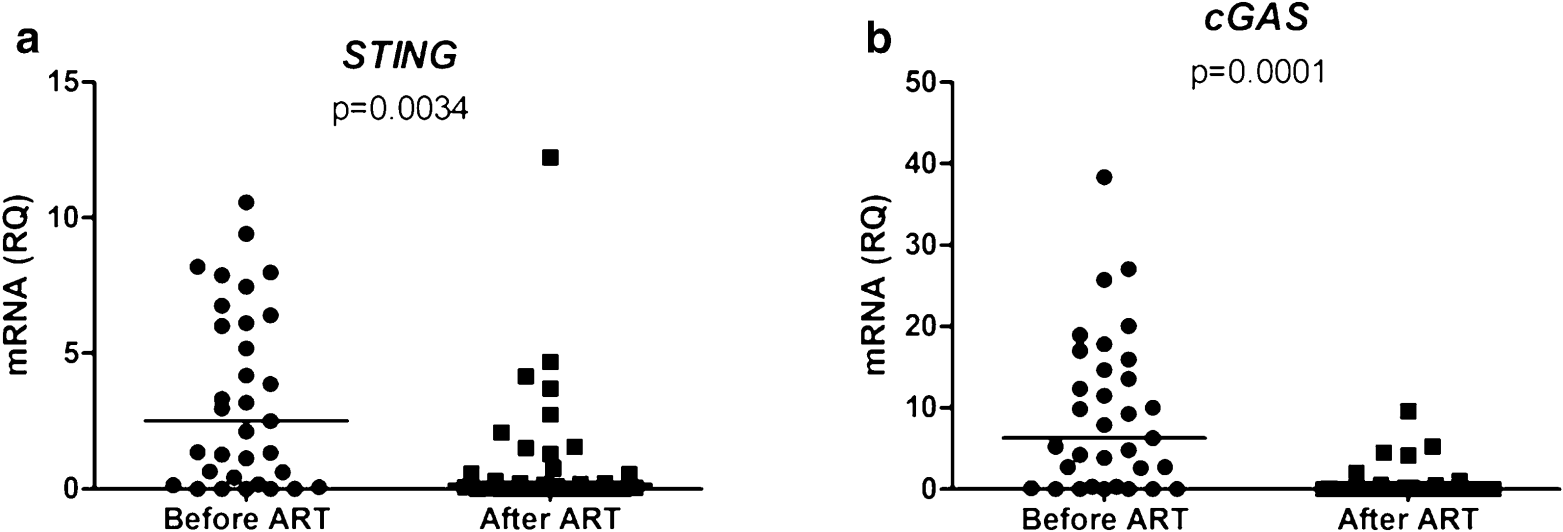
Comparison of mRNA levels of **a** STING and **b** cGAS before and after ART

The comparison of the mRNA levels of *STING* and *cGAS* between the periods evaluated showed that both genes were downregulated after 12 months of therapy. The median relative *STING* expression value decreased from 2.50 to 0.16 (p = 0.0034) (Fig. 2a). The median *cGAS* level decreased from 6.29 to 0 (p = 0.0001) (Fig. 2b).

**Fig. 3:**
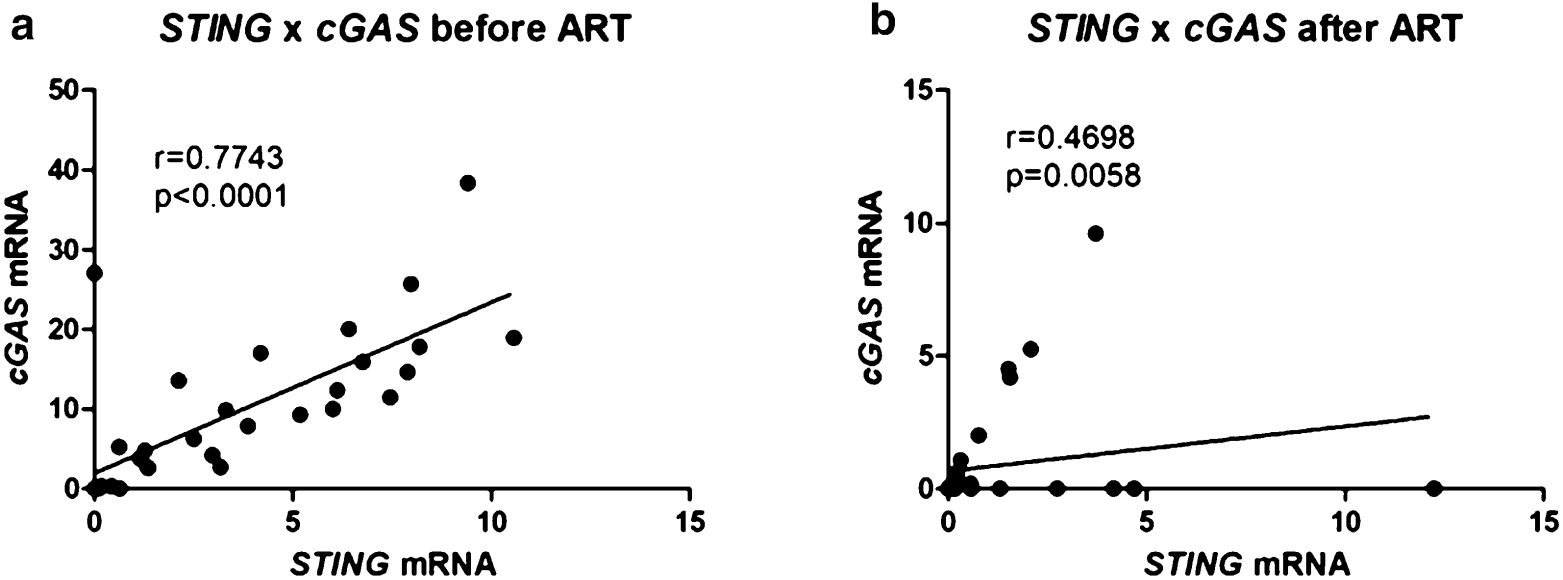
Correlation between STING and cGAS mRNA levels **a** before and **b** after the use of ART

The evaluation of the correlation between STING and cGAS mRNA levels showed a significantly positive correlation between the two markers before (p < 0.0001; Fig. 3a) and after the use of antiretroviral therapy (p = 0.0058, Fig. 3b).

**Fig. 4:**
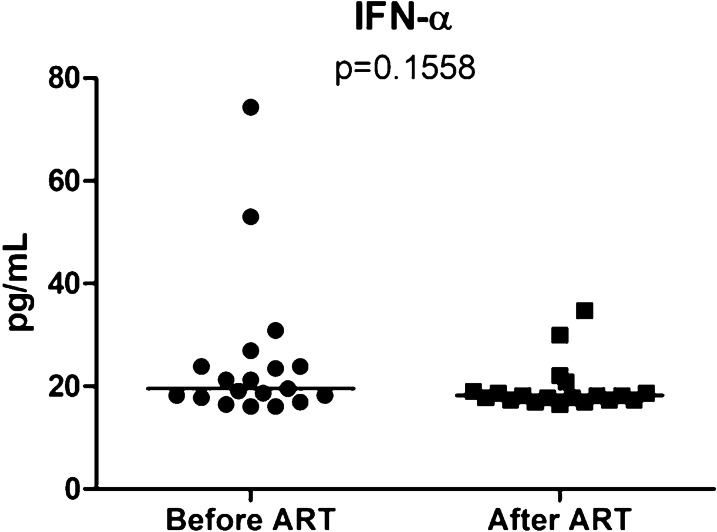
Comparison of plasma IFN-α level before and after the use of ART

The median plasma IFN-α level before ART was 19.57, which decreased slightly after 12 months of ART (18.26) (p = 0.1558) (Fig. 4).

**Fig. 5:**
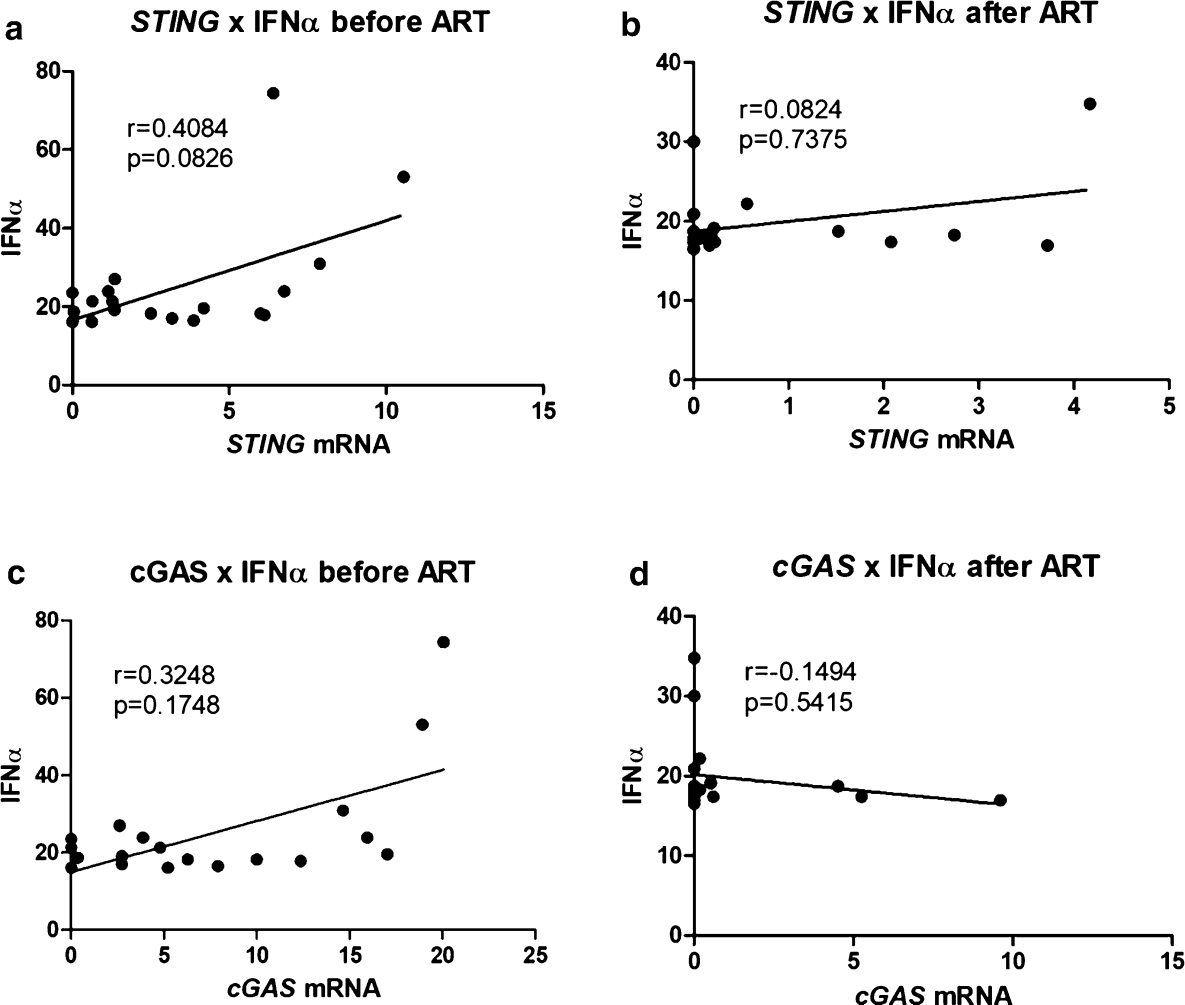
Correlation between IFN-α level and STING mRNA level **a** before and **b** after ART; correlation of IFN-α and cGAS levels **c** before and **d** after ART

The correlations between IFN-α level and *STING* and *cGAS* expression levels showed that there was a positive correlation trend for *STING* in both periods, but without statistical significance (Fig. 5a, b). For *cGAS*, the correlation with IFN-α showed a positive trend before the use of ART and a negative trend after its use (Fig. 5c, d). For a better understanding of the relationship between the levels of these markers before ART, the quadratic regression model was performed, which showed in more detail the distribution of the samples regarding to the levels of IFN-α and STING (r2 = 0.87, p < 0. 0001) and between IFN-α and cGAS (R2 = 0.68, p < 0.0001) (Additional file 1).

The correlation analyses of the *STING* and *cGAS* mRNA levels with the CD4^+^ and CD8^+^ T cell levels are shown in Table 2. Significance was only observed for the correlation between *STING* and CD4^+^ T cells, which was negative before treatment (p = 0.0463). The correlation between *STING* and CD8^+^ T cells remained positive in both analyses and was higher after the beginning of therapy (r = 0.1678; p = 0.3505). Regarding the CD4^+^/CD8^+^ T cell ratio, the expression of the *STING* gene showed a correlation that went from negative (r = −0.2687, p = 0.1306) to positive (r = 0.1270, p = 0.4812) after starting ART. For the *cGAS* × CD4^+^ T cell correlation analysis, r = −0.2740 and p = 0.1228 were obtained in the first collection, while the results were r = 0.2889 and p = 0.1030 at the second collection. The results of the correlation of *cGAS* expression with CD8^+^ T cell count showed a weak correlation before (r = −0.1523, p = 0.4133) and after treatment started (r = 0.1770, p = 0.3325). The correlation of *cGAS* with the CD4 + /CD8 + T cell ratio was not significant but went from negative in the first collection (r = −0.1824; p = 0.3096) to positive in the 2nd collection (r = 0.06033; p = 0.7388).

**Table 2:** Results of the correlation tests of STING and cGAS gene expression with the levels of CD4^+^ and CD8^+^ T cells and the CD4^+^/CD8^+^ T cell ratio before and after ART

	STING		cGAS	
	r	p value	r	p
*Before ART*				
CD4^+^ T cells	− 0.3493	0.0463	− 0.2740	0.1228
CD8^+^ T cells	0.0846	0.6398	− 0.1523	0.4133
CD4^+^/CD8^+^ T cell ratio	− 0.2687	0.1306	− 0.1824	0.3096
*After ART*				
CD4^+^ T cells	0.2408	0.1771	0.2889	0.1030
CD8^+^ T cells	0.1678	0.3505	0.1770	0.3325
CD4^+^/CD8^+^ T cell ratio	0.1270	0.4812	0.0603	0.7388

## Discussion

Since the advent of ART for people living with HIV, a significant reduction in AIDS-related morbidity and mortality has been observed. The best results are obtained from individuals who achieve immune recovery, which is mainly represented by the restoration of CD4^+^ T cell levels. Appropriate use of the therapy is also necessary for suppression of viral replication. Together, the beneficial effects of ART lead to a better clinical prognosis for patients [23, 24].

Studies evaluating the impact of ART are reasonable in relating the administration of therapy to the improvement of virologic and immunologic status and the reduction in the risk of AIDS progression. Such results have been observed since the introduction of ART in different countries and agree with the results obtained in the present study [25–28].

The effects of ART on innate immune markers are still not fully understood, as it is the case for the STING and cGAS molecules, which are important elements in the IFN-I production cascade that is responsible for antiviral action [11, 20]. Therefore, the study of these markers is useful to evaluate their roles in HIV-1 infection, as well as their impact before and after the use of ART. Our results show that *STING* and *cGAS* gene expression decreased after the use of ART. This result could be related to the reduction or abolition of viral replication induced by ART. A minimum amount of nucleic acid accumulation is necessary for activation of the cGAS-STING pathway [29], so if viral replication is inhibited by therapy, there will most likely be too little cDNA to induce the expression of these genes.

The activity of cGAS is essential for the detection of CDNs by STING in viral infection [15, 17]. The correlation between STING and cGAS levels showed that before therapy there was a greater positive correlation than after the use of ART, showing that the infection induces the continuous expression of the two restriction factors for activation of innate immunity mechanisms against HIV-1 infection, while the use of HAART influenced the reduction in the levels of factors, which resulted in a reduction in the correlation between both.

Similar to what was observed in our study, Nissen et al. (2014) also reported higher levels of *cGAS* expression in individuals who did not use ART [30]. Reverse transcriptase inhibitors promote *cGAS* inhibition because they inhibit the formation of viral DNA which is crucial for cGAS activity [21]. These results show that a high HIV-1 replication rate contributes to *cGAS* gene expression. The evaluation of the impact of ART in an Ugandan cohort showed a downregulation of several antiviral response genes after starting ART, including *IRF7* and *OAS1*, a gene which protein has structural and functional homology with *cGAS* [31]. In a similar analysis, Li et al. (2004) also found a reduction in the expression of 26 genes after the use of ART, which, like *STING* and *cGAS*, were related to IFN production [32]. In this sense, the present study corroborates previous information that ART acts as a downregulator of *STING* and *cGAS* since it significantly reduces the levels of PAMPs detected by the pathway.

The activity of IFN-I in the control of viral infections is a point already widely discussed in the literature. Cytokines are considered key effector molecules in the innate immune response and have widespread effects and the ability to quickly stimulate the entire immune system [33]. In viral infections, the main molecular patterns are nucleic acids, which are a strong stimulator of the IFN-I response [10].

The activity of IFN-α consists of promoting an antiviral state in the host cell through restriction factors that prevent viral replication and by stimulating other immune response cells, such as natural killer cells [34]. IFN-α also contributes to a sustained immune activation and exacerbated inflammatory response, making for an important duality for this cytokine and making its role possibly controversial in some cases [35]. In the present study, plasma levels of IFN-α were slightly lower after the use of ART, but not significantly. This may be explained by the activity of other pathways acting in a cGAS-STING- independent manner, sensors such as RIG-I [36] or TLRs (TLR7, TLR9) [10, 37] that might not have been strongly affected by ART. The production of type 1 interferons, including IFN-α, in the immunopathogenesis of HIV-1, in addition to being related to the antiviral response, can induce inflammatory mechanisms by persistent immune activation and even T-cell exhaustion [35, 38], which may contribute to the progression to AIDS.

Studies such as by French et al. (2009) and Malherbe et al. (2014) found that even with virologic success and immune recovery, IFN-α did not undergo a significant reduction after the onset of ART [39, 40]. This finding agrees with ours and, together, may suggest that the production of IFN-α after the use of ART is more related to immune activation than to antiviral activity. Therefore, it is possible that the use of antiretroviral therapy is not sufficient to completely abolish the production of immune activators as inflammatory mediators, even with therapeutic success in reducing the viral load and recovering CD4^+^ T cells.

The positive correlation observed between the expression of *STING* and *cGAS* and the levels of interferon can be explained by the function of the *STING* and *cGAS* genes, which are, respectively, an adapter and a sensor of innate immunity, parts of an important cascade that results in the production of IFN-I against viral infections, among other stimuli. With the availability of viral load for detection by the sensors, high expression of these genes and high IFN-I levels were expected in the evaluation before the start of ART [21, 41, 42]. The present study, after starting ART, IFN-α did not follow the same pattern of significance as *STING* and *cGAS*. A possible explanation for this may be the activity of another gene stimulated by interferon, such as *Mx2* [43], *IRF1* [44], *Viperin*, or the *IFIT1*, *-2*, or *3* gene [45], which were not evaluated in the present study.

The negative correlation observed between *STING* and CD4^+^ T cell level can be explained by understanding the immunological characteristics of acute HIV-1 infection without treatment. Cerboni et al. (2017) suggested that *STING* plays a downregulator role in the proliferation of T lymphocytes [46]. The evaluation of the expression of *STING* and *cGAS* with the levels of the other immune response markers (CD8^+^ T cells and CD4^+^/CD8^+^ T cell ratio) suggests that these are not directly correlated. However, *STING* and *cGAS* have been associated with increased stimulation of responses by CD8^+^ T cells [47, 48]. Mechanistic studies should be performed to better elucidate this relationship.

## Conclusions

The use of ART provided immune recovery and viral suppression to the studied group and indirectly induced the downregulation of *STING* and *cGAS*. In contrast, ART did not affect plasma IFN-α. The expression of *STING* and *cGAS* were not correlated with plasma IFN-α, which suggests that the STING–cGAS pathway is not the main cytokine-inducing pathway.

## Supplementary Information

**Additional file 1.** Quadratic regression model showing in more detail the distribution of the samples in relation to the levels of (A) IFN-alpha and STING (r2 = 0.87, P < 0.0001) and between (B) IFN-alpha and cGAS (R2 = 0.68, P < 0.0001), before ART.

## Data Availability

Data are available from the corresponding author upon reasonable request.

## Abbreviations

AIDS: Acquired immunodeficiency syndrome
ART: Antiretroviral therapy
cAMP: Cyclic adenosine monophosphate
cGAS: CGMP-AMP synthase
cGMP: Cyclic guanosine monophosphate
c-di-AMP: Cyclic dimeric adenosine monophosphate
ci-di-GMP: Cyclic dimeric guanosine monophosphate
cGAMP: Cyclic guanosine monophosphate–adenosine monophosphate
cDNA: Complementary DNA
CDN: Cyclic dinucleotide
CT: Cycle treshold
dsDNA: Double-stranded DNA
HIV-1: Human immunodeficiency virus 1
IFN: Interferon
IRF: Interferon regulatory factor
mRNA: Messenger RNA
NF-κB: Nuclear factor κB
OAS1: Oligoadenilato sintetase 1
PAMP: Pathogen-associated molecular pattern
qPCR: Quantitative polymerase chain reaction
RIG-I: Retinoic acid-inducible gene I
STING: Stimulator of interferon genes
