# Supplementary figures and images for: STING and cGAS gene expressions were downregulated among HIV-1-infected persons after antiretroviral therapy
Source: Virol J. 2021 Apr 15;18:78. doi: 10.1186/s12985-021-01548-6 (PMC8047565; doi:10.1186/s12985-021-01548-6)

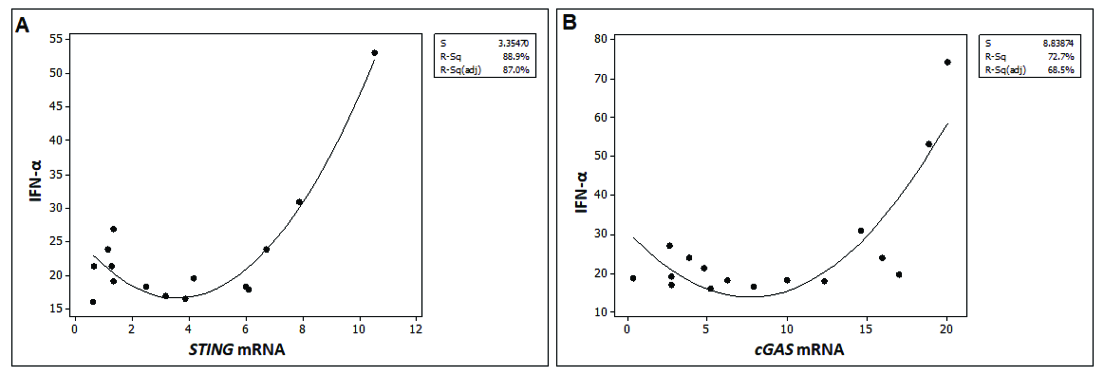

Supplement: Supplementary file 1 — Additional file 1. Quadratic regression model showing in more detail the distribution of the samples in relation to the levels of (A) IFN-alpha and STING (r2 = 0.87, P < 0.0001) and between (B) IFN-alpha and cGAS (R2 = 0.68, P < 0.0001), before ART. [file 12985_2021_1548_MOESM1_ESM.tif]
